# Unveiling the Invisible: Complication of Laparoscopic Sacrocolpopexy Illuminated by Transcervical Injection of ICG

**DOI:** 10.1007/s00192-025-06148-4

**Published:** 2025-04-24

**Authors:** Giovanni Ruggeri, Anna-Sophie Villiger, Michael David Mueller, Annette Kuhn

**Affiliations:** Department of Obstetrics and Gynecology, University of Bern University Hospital, Friedbühlstrasse 19, 3010 Bern, Switzerland

**Keywords:** Sacrocolpopexy, Comlication, ICG, Laparoscopy, Pelvic Organ Prolaps

## Abstract

**Introduction:**

A 57-year-old patient was referred to our tertiary referral urogynecology unit due to persistent and profuse vaginal discharge 1 year after undergoing laparoscopic sacrocolpopexy with subtotal hysterectomy for apical prolapse. During clinical examination, abundant yellowish discharge from the cervix was observed. Creatinine testing and Uro-CT excluded vesicovaginal or ureterovaginal fistulas and large abscesses. However, owing to suspected infection and the patient’s poor quality of life, a decision was made to proceed with laparoscopic revision.

**Results and Methods:**

Intraoperatively, transcervical injection of ICG (indocyanine green) successfully illuminated a hidden retroperitoneal sinus and a low-volume abscess extending to the sacral promontory. Therefore, the previously installed mesh and the cervix were removed in a challenging but complication-free procedure. The patient was followed up after 3 months, remaining asymptomatic and satisfied with the outcome.

**Conclusions:**

Managing complications following laparoscopic sacrocolpopexy requires thorough clinical and instrumental evaluation. In this case, the strategic use of ICG injection proved to be an innovative approach to visualizing hidden complications, offering valuable insights for handling complex scenarios.

**Supplementary Information:**

The online version contains supplementary material available at 10.1007/s00192-025-06148-4.

## Introduction

Sacrocolpopexy is the preferred approach for treating apical prolapse due to its favorable patient satisfaction rates and low re-operation rates [1, 2] in specific situations. However, mesh-related complications are increasingly a concern, as the foreign material can erode, cause pain, or become infected. Reported mesh complication rates range from 0 to 5% [2]. Mesh-related infections are uncommon in pelvic organ prolapse repairs, occurring in 0.3% of laparoscopic sacrocolpopexy cases [3] and 0.7–0.8% of vaginal mesh placements [4]. The standard treatment for mesh-related infections is often mesh resection. The aim of this video is to demonstrate the process leading to mesh removal using the strategic application of indocyanine green (ICG).

## Materials and Methods

This video demonstrates the laparoscopic management of mesh excision, accompanied by narrated images. A 57-year-old female patient was referred to our urogynaecology unit because of persistent, profuse vaginal discharge for 1 year following laparoscopic sacrocolpopexy with subtotal hysterectomy for the treatment of apical prolapse. Clinical examination revealed a yellowish discharge from the cervix. To rule out possible causes, creatinine quantification of the fluid and a uro-CT scan were performed, both of which excluded the presence of vesicovaginal or ureterovaginal fistulas and major abscesses. Bacterial culture of the secretion was performed and showed no bacterial growth. Despite the absence of these common complications, the patient’s persistent symptoms and decreased quality of life raised a strong clinical suspicion of a mesh-related infection. The decision was made to perform a laparoscopic revision using a transcervical injection of 10 ml indocyanine green (ICG) to improve visualization during surgery. The transcervical ICG injection illuminated a retroperitoneal sinus formation and an abscess. These findings extended to the sacral promontory. On the basis of these observations, the previously implanted mesh was removed along with the remaining cervix. The procedure, although technically challenging, was completed without complications. Regarding the bladder lesion mentioned in the video, this was identified intraoperatively by direct visualization and sutured with a PDS 3.0 suture. A thorough cystoscopic examination was also performed during the procedure, confirming the integrity of the bladder after repair. Post-operative follow-up visits at 3 and 6 months revealed that the patient was asymptomatic and highly satisfied with the surgical outcome, reporting no recurrence of her symptoms.

## Discussion

Laparoscopic sacrocolpopexy is widely considered the gold standard for advanced prolapse repair due to its efficacy and favorable long-term results [5]. Although mesh complications are relatively rare, when they do occur, one in ten require interventions such as mesh removal.

The increasing use of synthetic mesh in urogynaecological and pelvic surgery has led to a parallel increase in mesh-related complications [6, 7]. Reported complications include mesh erosion (11%), mesh exposure (10.5%), infection (0.7–8%), recurrent prolapse (21% anatomical and 10.5% symptomatic), dyspareunia (9%), wound granulation (7.8%), and more serious problems such as organ perforation or bowel obstruction (6%), bladder and ureteral injury (< 3%) [6].

The procedure used a monofilament macroporous polypropylene mesh as required for mesh in prolapse surgery. The sutures used were Ethibond multifilament sutures. As the procedure was originally performed in a remote hospital, we can only speculate that these materials were chosen based on availability and the specific surgical context. However, we acknowledge the potential risks associated with multifilament, nonabsorbable sutures, such as increased bacterial colonization, which could lead to complications such as fistula or abscess formation. Recent literature has examined the impact of suture selection in sacrocervicopexy. A randomized controlled trial comparing absorbable and permanent sutures in laparoscopic sacrocervicopexy found that while both types of sutures provided effective results, nonabsorbable sutures provided more durable fixation but carried a potential risk of infection-related complications [9]. In terms of recommendations, monofilament nonabsorbable sutures such as polypropylene may be a viable alternative as they have a lower risk of bacterial adherence while maintaining long-term support. Ultimately, the choice of suture should be based on the patient’s specific conditions, the surgeon’s expertise, and institutional protocols.

Complete removal of the mesh was deemed necessary in this case due to the presence of persistent and profuse vaginal discharge, which significantly impaired the patient’s quality of life. The infection had spread to the entire area of the mesh, necessitating its complete removal, according to the 2017 ACOG Committee Opinion “Management of Mesh and Graft Complications in Gynaecologic Surgery,” which recommends complete mesh removal in the presence of infection to ensure resolution of symptoms and prevent further adverse outcomes [8]. Retaining the mesh under such conditions would have posed significant risks, including chronic infection, further tissue damage, and systemic complications. The use of transcervical injection of indocyanine green (ICG) in this case represents a novel approach in our clinical practice. While we have experience with ICG in other gynecological procedures, such as identifying ureteral patency or improving lymph node visualization, this was one of our first applications of ICG to detect complications related to sacrocolpopexy mesh. In this particular case, we believe that ICG injection significantly improved our ability to identify the retroperitoneal abscess. Although clinical suspicion and symptoms such as persistent cervical discharge strongly suggested a mesh-related infection, the anatomical extent of the infection and the precise location of the abscess may have been more difficult to delineate without ICG.

## Conclusion

Management of complications after laparoscopic sacrocolpopexy requires careful clinical and instrumental evaluation. In the presented case, the strategic use of ICG injection emerged as an innovative approach to visualize hidden complications. This case underscores the utility of early diagnosis and intervention, offering valuable insights for difficult scenarios.

## Supplementary Information

Below is the link to the electronic supplementary material.

Supplementary file 1 (MP4 305092 KB)

## Data Availability

No new data were generated or analyzed in this study.
